# Culturally Adapted Guided Internet-Based Cognitive Behavioral Therapy for Hong Kong People With Depressive Symptoms: Randomized Controlled Trial

**DOI:** 10.2196/64303

**Published:** 2025-02-25

**Authors:** Jia-Yan Pan, Jonas Rafi

**Affiliations:** 1 Department of Social Work Hong Kong Baptist University Hong Kong China (Hong Kong); 2 Department of Psychology Stockholm University Stockholm Sweden

**Keywords:** Internet-based cognitive behavioral therapy, depression, Chinese, Hong Kong, culturally adapted internet intervention

## Abstract

**Background:**

A significant number of individuals with depression in Hong Kong remain undiagnosed and untreated through traditional face-to-face psychotherapy. Internet-based cognitive behavioral therapy (iCBT) has emerged as a tool to improve access to mental health services. However, iCBT remains underdeveloped in Chinese communities, particularly in Hong Kong.

**Objective:**

This study aims to (1) develop and evaluate the effectiveness of a culturally and linguistically appropriate guided iCBT program for Hong Kong Chinese with depressive symptoms, and (2) explore their treatment adherence.

**Methods:**

An 11-week guided iCBT program, “Confront and Navigate Depression Online” (CANDO), consisting of 8 online modules, was developed and implemented for Hong Kong residents. The program was accessible via either an online platform (web-based iCBT) or a smartphone app (app-based iCBT). A 3-arm randomized controlled trial was conducted, with participants recruited through open recruitment and referrals from 2 local non-governmental organizations. A total of 402 eligible participants with mild to moderate depressive symptoms were randomly allocated into 3 groups: (1) web-based iCBT (n=139); (2) app-based iCBT (n=131); and (3) waitlist control (WLC) group (n=132), who transitioned to the web-based iCBT group after waiting for 11 weeks. Therapist support was provided by a clinical psychologist through 3 counseling sessions and weekly assignment feedback. The primary outcomes were the Beck Depression Inventory-II (BDI-II) and the 9-item Patient Health Questionnaire (PHQ-9), while the secondary outcome measures included the 12-item General Health Questionnaire (GHQ-12), the Chinese Automatic Thoughts Questionnaire (CATQ), and the Chinese Affect Scale (CAS). These scales were administered at preintervention, postintervention, and at 3-month and 6-month follow-up assessments. Data analysis was conducted using linear mixed effects modeling, adhering to the intent-to-treat principle.

**Results:**

Participants in both the web- and app-based iCBT groups reported significant improvements compared with the WLC group on all primary (*P*<.001) and secondary measures (*P*<.001 and *P*=.009) at posttreatment. Large between-group effect sizes were observed in the reduction of depressive symptoms, with Cohen's d value of 1.07 (95% CI 0.81-1.34) for the web-based group and 1.15 (95% CI 0.88-1.43) for the app-based group on the BDI-II. Additionally, the web- and app-based groups showed effect sizes of 0.78 (95% CI 0.52-1.04) and 0.95 (95% CI 0.63-1.27) on PHQ-9, respectively. Medium to large effect sizes were observed for secondary outcomes at posttreatment. These positive effects were maintained at both the 3- and 6-month follow-ups, with medium to large within-group effect sizes. The adherence rate in the 2 iCBT groups was 154 out of 270 (57.0%) for completing all 8 online modules and 152 out of 270 (56.3%) for attending all 4 counseling sessions (including an intake interview). The recovery rate, as measured by the BDI-II at posttreatment, was 35 out of 90 (39%) for the web-based group and 36 out of 91 (40%) for the app-based group, compared with 3 out of 112 (3%) in the WLC group.

**Conclusions:**

Culturally and linguistically adapted iCBT is an effective and feasible treatment for Hong Kong Chinese with mild to moderate depressive symptoms, demonstrating a satisfactory recovery rate and treatment adherence. ICBT offers an accessible and viable alternative to face-to-face interventions in Hong Kong. The Hong Kong government should allocate more resources and support the use of iCBT as a tool to treat people with depressive symptoms.

**Trial Registration:**

ClinicalTrials.gov (NCT04388800); https://clinicaltrials.gov/study/NCT04388800

## Introduction

### Background

Depression is a widespread mental health issue that the World Health Organization (WHO) predicts will become the most burdensome globally by 2030 [1]. In Hong Kong, the prevalence of depression rose from 1.3% in 2011-2014 to 9.1% in 2019 among the general population [2]. Lam et al [3] reported a 13.3% prevalence of common mental disorders in Hong Kong, with mixed anxiety and depression being the most frequent diagnosis (6.9%). However, only 24%-26% of individuals with common mental disorders access mental health services [3]. Consequently, many people in Hong Kong, similar to those with mental health conditions in Western countries [4], remain undiagnosed and untreated. This is likely due to the stigma surrounding mental disorders, a shortage of mental health professionals, the high cost of private treatment, and long wait times for public services. The lack of treatment not only leads to poor mental health outcomes, such as the development of comorbid anxiety and substance use disorders, as well as an increased risk of suicide [5], but also manifests physically, including chronic pain [6], thereby reducing quality of life [5]. Furthermore, poor mental health outcomes have significant economic consequences, such as reduced productivity [7]. These factors highlight the critical importance of early diagnosis and treatment.

### Internet-Based Cognitive Behavioral Therapy

Cognitive behavioral therapy (CBT) is the first-line treatment for depression. Andersson and Titov [8] demonstrated that internet-based CBT (iCBT) produces treatment effects comparable to traditional face-to-face CBT, while being more cost-effective. As Baumann et al [9] stated, “ICBT has a strong potential to balance demand and supply of CBT in unipolar depression by reducing therapist time per patient.”

Karyotaki et al [10] systematically reviewed 39 randomized controlled trials (RCTs) and found that both guided and unguided iCBT interventions are more effective than control treatments (treatment-as-usual and waitlist control [WLC]) in reducing depressive symptom severity in both the short and long term. Etzelmueller et al [11] conducted a meta-analysis demonstrating the acceptability and effectiveness of guided iCBT for treating depression in routine health care. While guided iCBT is more effective than unguided iCBT at posttreatment, no differences are observed at 6- or 12-month follow-ups [10].

Mobile therapy has gained prominence with the widespread adoption of smartphones, bringing mobile psychotherapy to the forefront. Studies indicate no significant differences in treatment effectiveness between guided smartphone-delivered and internet-based CBT programs [12]. However, smartphone use is more evenly distributed throughout the day, allowing iCBT to integrate more seamlessly into daily routines [12]. Given the high penetration rates of smartphones (97%) and computers (89%) in Hong Kong [13,14], developing an iCBT program accessible via both mobile apps and computers—and comparing their effectiveness—would be a valuable endeavor.

In recent years, particularly during the COVID-19 pandemic, iCBT has seen rapid development in Chinese societies. Studies have shown that culturally adapted iCBT significantly reduces symptoms of depression, generalized anxiety, psychological distress, rumination, and insomnia while improving self-efficacy among Chinese individuals, compared with WLCs [15-18]. Even in studies without WLCs, culturally adapted iCBT has proven effective in reducing symptoms of depression, generalized anxiety, psychological distress, perceived stress, sleep disturbances, and functional disability, while enhancing life satisfaction and energy levels for Chinese participants [19,20]. The positive effects of culturally adapted iCBT were sustained for 3 months [19], 6 months [18], and 12 months [15]. These studies used RCTs with either an active control group (eg, [21]) or a WLC group (eg, [16,18]). Most studies utilized mobile interventions (eg, [16-18]), while a few relied on web-based interventions [15,19], and 1 incorporated both [20]. To better address the diverse mental health needs of the Chinese population, further research on culturally adapted and tailored iCBT programs is needed.

Response rates of iCBT for depression, indicating a significant reduction in depressive symptoms, range from 37% to 56% in meta-analyses [10,22]. The response rate for guided iCBT (48%) is higher than that for unguided iCBT (37%) [10] and significantly higher than that of control groups [23]. Recovery rates of guided iCBT for depression, defined as the complete remission of depressive symptoms, have been reported to range from 30% to 55% at posttreatment and 41% to 60% at the 6- to 7-month follow-up [24,25]. Lin et al [16] found that the guided iCBT program for Chinese individuals with depression achieved a high response rate (58%) and remission rate (55%). Similarly, Ying et al [18] reported that 38.2% and 33.3% of Chinese participants in their iCBT study demonstrated clinically meaningful improvements in depressive symptoms at postintervention and at the 6-month follow-up, respectively.

Regarding treatment adherence to iCBT, van Ballegooijen et al [26] found that the completion rate of the entire guided iCBT program for depression was 65.1%, significantly lower than the rate for face-to-face CBT (84.7%). However, there was no significant difference in the number of completed sessions, with 83.9% in face-to-face CBT and 80.8% in guided iCBT [26]. Similarly, Karyotaki et al [10] reported in their meta-analysis an average adherence rate (number of completed sessions/total number of sessions) of 76% for guided iCBT and 54% for unguided iCBT. Individual empirical studies show that the adherence rate (percentage of completers) of guided iCBT for depression is higher among a Chinese population, ranging from 67.0% [21] to 81.4% [18], compared with their Western counterparts, whose adherence rates range from 36.4% [27] to 60% [28]. The average session completion rate in guided iCBT for Hong Kong Chinese ranges from 52.7% [21] to 83.8% [18]. Karyotaki et al [10] also reported that the average dropout rate was 25% for guided iCBT and 29% for unguided iCBT, compared with 19% for those on a WLC and 22% for those receiving treatment as usual. Additionally, Chinese participants in iCBT reported high levels of satisfaction during depression treatment (eg, [16,18]).

### Cultural Adaptation of iCBT

There is evidence suggesting the potential benefits of cultural adaptation in digital mental health interventions, including iCBT, for improving a range of outcomes compared with WLC and treatment-as-usual control conditions [29]. However, direct comparisons of the effectiveness of culturally adapted versus unadapted internet-based interventions remain lacking. For Chinese participants, Ng and Wong [30] demonstrated that the short-term effects of culturally adapted CBT were stronger than those of unadapted CBT. Similarly, iCBT should be culturally adapted to address the specific cultural needs of Chinese clients, taking into account cultural factors that may influence its effectiveness and acceptability. The following considerations are important when promoting iCBT to Chinese clients. First, the program should use culturally appropriate language, including Mandarin or other Chinese dialects, as well as idioms and expressions, to enhance understanding and engagement. For instance, Wong [31] translated the term “cognitive distortion” into the Chinese phrase “Xi Xiang Xian Jing (思想陷阱)” and adapted various types of cognitive distortions into 4-character Chinese idioms. Second, cultural sensitivity and awareness should be incorporated into the program content to align with Chinese cultural norms, values, and experiences. For example, an iCBT program for Hong Kong Chinese university students integrated Chinese cultural values into its content [32]. Additionally, collectivism should be emphasized by addressing relationships with others, social role expectations, and family obligations, which are highly valued in Chinese culture [33]. Third, the stigma surrounding mental health issues often deters Chinese individuals from seeking face-to-face services [34]. ICBT programs should tackle this cultural stigma by providing psychoeducation on mental health and emphasizing the importance of help-seeking. Fourth, when designing an iCBT program for Chinese participants, incorporating more videos (eg, case studies and animations) and reducing text can foster greater interaction and engagement [32,33]. Finally, therapists in guided iCBT programs should possess cultural and linguistic competence in Chinese culture and language [32].

Undoubtedly, iCBT is an effective, feasible, and acceptable approach for treating depression among Chinese individuals. However, it remains underdeveloped in Chinese communities, particularly in Hong Kong. Moreover, whether the delivery medium (web- or app-based) influences treatment outcomes is still unclear. To address this, this study developed and tested the effectiveness of a culturally and linguistically appropriate guided iCBT program for Hong Kong people with mild to moderate depressive symptoms, utilizing both web- and app-based platforms. We hypothesized the following:

- Participants in the web- and app-based iCBT groups would have lower levels of depressive symptoms upon completion of the intervention compared with the WLC group.
- Participants in the web- and app-based iCBT groups would have lower levels of anxiety symptoms, psychological distress, and negative thoughts and emotions upon completion of the intervention compared with the WLC group.
- Participants in the web- and app-based iCBT groups would have higher levels of positive thoughts and emotions upon completion of the intervention compared with the WLC group.
- There would be no significant differences in intervention effects between the web- and app-based iCBT groups.
- The effects of the web- and app-based iCBT programs would be sustained at 3 and 6 months postintervention.

## Methods

### Participant Selection Criteria

Participants were selected based on the following criteria: (1) aged 18-70 years; (2) proficient in Chinese; (3) a 9-item Patient Health Questionnaire (PHQ-9) score between 5 and 19; (4) no suicidal risk in the past 3 months, defined as a score of “0” or “1” on Item 9 of the Beck Depression Inventory-II (BDI-II) and low suicidal risk as assessed during the intake interview; (5) not receiving any other psychological treatment for depression at the time of allocation; (6) no severe psychiatric conditions, such as bipolar disorder, as self-reported in the screening questionnaire and confirmed by the therapist during the intake interview; (7) access to a computer or smartphone with an internet connection; and (8) possession of a valid email address.

### Recruitment and Screening

Participants were recruited from the general population in Hong Kong on an ongoing basis from April 2020 to January 2022. Recruitment methods included advertisements on social media platforms (Facebook and Instagram), Google Ads, a press conference, posters, leaflets, and referrals from 2 local partner nongovernmental organizations. Interested individuals registered at [35] by completing a screening questionnaire that included the PHQ-9, the BDI-II, and the Beck Anxiety Inventory (BAI). Initially, qualified participants were invited to a 1.5-hour intake interview conducted in-person or via Zoom (Zoom Communications/Qumu Corporation) with a clinical psychologist or an experienced social worker to further assess their suitability for participation, including a suicidal risk assessment. Marginal cases were discussed in a case meeting that included the first author (JYP), intake worker, and program therapist to assess participation eligibility. High-risk individuals with severe depression or suicidal risk or both were referred to local mental health services for further diagnosis and treatment with their consent.

### Research Design

A 3-arm RCT was conducted with a WLC group design. Randomization was performed using computer-generated random numbers by the therapist to allocate eligible participants to 1 of 3 groups: web-based iCBT, app-based iCBT, and WLC (1:1:1 allocation). Participants in the WLC group received web-based treatment immediately after the 2 iCBT groups completed their program. All 3 groups completed the same online questionnaire at pretest, posttest, and at 3- and 6-month follow-ups. The WLC group completed the questionnaire an additional time as a posttest after the 2 experimental groups had completed the program.

### Ethical Considerations

An informed consent form was presented at the beginning of the questionnaire. After participants provided their consent to join the study, the online questionnaire was displayed to them. The RCT was conducted with a total of 13 cohorts from May 2020 to January 2023, with around 30 qualified participants in each cohort for implementation feasibility. The program evaluators were blinded to participant allocation. Ethics approval was obtained from the Research Ethics Committee of the university of the first author (JYP). The clinical trial was registered at ClinicalTrials.gov (NCT04388800).

### Sample Size

A power analysis was conducted to calculate the required number of participants, assuming a medium effect size (*d*) of 0.5 [16], an α level of .05, and 95% power. The minimum required sample size was estimated to be around 210 participants. Given an assumed completion rate of 55%, the goal was to recruit approximately 400 participants.

### Intervention: Confront and Navigate Depression Online (CANDO) Program

The “Confront and Navigate Depression Online” (CANDO) Program was an 11-week therapist-guided iCBT program designed for Hong Kong Chinese with mild to moderate depressive symptoms (Multimedia Appendix 1). The program was developed based on the principles of CBT as outlined by Beck [36], and adapted for Chinese clients (eg, [31,37]). It utilized a blended mode of service delivery, featuring 8 online modules and four 1-hour individual counseling sessions including 1 intake interview. Each module introduced different cognitive behavioral skills for coping with depression through self-developed videos. The themes of the 8 online modules are (1) understanding my depression within the CBT framework; (2) identifying automatic thoughts and cognitive distortions; (3) self-talk and positive statements; (4) behavioral experiments and behavioral activation; (5) problem-solving; (6) identifying and understanding cognitive rules; (7) relaxing dysfunctional cognitive rules; and (8) conclusion and relapse prevention. A variety of CBT skills were introduced and reinforced using animated videos and demonstrated through 5 local cases. Each online module began with a mood check for the past week using a 10-point scale and concluded with a session review and an assignment designed to help clients apply the skills they had learned to manage their depression. The modules were released to participants on a weekly basis, with counseling sessions scheduled in between for skills application. Additional program features included a client portfolio, online questionnaires, reminders, and an online booking system.

The development of the CANDO Program was guided by the cultural adaptation framework for psychosocial treatments proposed by Bernal et al [38]. The components listed in Textbox 1 were addressed to culturally adapt the program for Hong Kong Chinese.

**Components addressed to culturally adapt the program for Hong Kong Chinese.**

**1. Language**

The entire program was developed and delivered in the local Cantonese language.

**2. Metaphors**

Various cognitive distortions were represented using 4-character Chinese idioms, such as “Fei Hei Ji Bai (非黑即白)” (all-or-nothing thinking), “Da Nan Lin Tou (大難臨頭)” (catastrophizing), and “Cai Duo Ren Yi (猜度人意)” (mind-reading) [31].

**3. Content**

The case demonstration videos were specifically tailored for Hong Kong Chinese people, drawing on and adapting numerous real-life cases within the Hong Kong context. These videos incorporated Chinese cultural values such as gender and social role expectations, face-saving, collectivism, and the high emphasis on academic achievement, addressing these themes across different life stages. For instance, an analysis of advantages and disadvantages was used to challenge and relax the cognitive rule of “男主外, 女主內” (“The husband is responsible for earning a living to support his family, while the wife is responsible for taking care of the family”) into a more progressive belief that “there is no fixed stereotype of gender roles within a family as society advances.” Another example is the use of a pie chart technique to redefine the concept of “a good mom” in Chinese society, broadening it to include multiple perspectives rather than focusing solely on helping children achieve academic excellence.

**4. Persons**

Therapists with the same ethnic and cultural background as the Hong Kong Chinese clients were selected for service delivery.

**5. Method**

The internet-based cognitive behavioral therapy delivery format was adapted to primarily use self-developed videos to teach and demonstrate various cognitive behavioral therapy skills.

The client platform offered both a web-based version, accessible via the program website, and a mobile app, available for download through Google Play (Google LLC/Alphabet, Inc.) or Apple Store (Apple, Inc.). After enrollment, clients were provided with an account to access the system. Clients with a PC web account could not log-in via the app, and those with an app account could not access the web-based version. Each client was assigned a therapist who provided therapeutic support through 3 counseling sessions conducted either face-to-face or online (via Zoom, WhatsApp call [Meta Platforms, Inc.], or telephone). These sessions included feedback on assignments, responses to internal messages, and online forum management. Both automated reminders and staff reminders were used to ensure participants remained engaged with the program.

To ensure data security and privacy, 2-factor authentication was implemented. This required users to log-in with a password preset by the user and a 1-time password sent to their email address. All collected electronic data were encrypted. The program was hosted on the server managed by the Information and Technology Office of the first author’s (JYP) university. Before the program’s launch, a user acceptance test was conducted to address bugs, and a security test was carried out by a third party to ensure system safety. Bugs reported by participants during the project were also addressed promptly throughout its implementation.

### Therapist Credibility

The therapist was a clinical psychologist with a doctorate in clinical psychology and a master’s degree in counseling. She had over 10 years of clinical experience in Hong Kong and the United States. The intake worker was a registered social worker in Hong Kong with bachelor’s and master’s degrees in social work, and more than 30 years of counseling experience in diverse settings. The intake interviews were conducted by both the therapist and the intake worker, while the initial screenings were performed by the therapist. All services were delivered in Cantonese, and both professionals were native Cantonese speakers.

### Measurements

Depressive symptoms were assessed using both the BDI-II [39] and PHQ-9 [40]. For the BDI-II, scores of 0-9, 10-18, 19-29, and 30 or above indicate minimal, mild, moderate, and severe levels of depressive symptoms, respectively. For the PHQ-9, scores of 0-4, 5-9, 10-14, 15-19, and 20-27 correspond to minimal, mild, moderate, moderately severe, and severe depression, respectively. Anxiety symptoms were evaluated using the BAI [41]. In this study, the Cronbach α values were 0.866 (95% CI 0.844-0.884) for the BDI-II, 0.812 (95% CI 0.779-0.839) for the PHQ-9, and 0.909 (95% CI 0.890-0.923) for the BAI, demonstrating high internal consistency for all measures.

Psychological distress was measured using the Chinese version of the 12-item General Health Questionnaire (GHQ-12) [42,43]. The 0-0-1-1 scoring method was applied to calculate the total score, which ranged from 0 to 12, with higher scores indicating greater levels of psychological distress. A cut-off point of 1/2, validated for the Chinese population, was used to identify participants at risk of developing mental health problems [44]. In this study, the Cronbach α for the GHQ-12 was 0.866 (95% CI 0.844-0.884), indicating high internal consistency.

Automatic thoughts were measured using the Chinese Automatic Thoughts Questionnaire [45], which was developed from the Automatic Thoughts Questionnaire [46] and the Positive Automatic Thoughts subscale of the revised Automatic Thoughts Questionnaire [47]. Participants rated the frequency of each thought in the past week on a 5-point scale (1=“Not at all” to 5=“All the time”). Item scores were averaged for the positive and negative subscales, with higher total scores indicating more positive and negative automatic thoughts, respectively. Cronbach α was 0.883 (95% CI 0.825-0.889) for the positive subscale and 0.88 (95% CI 0.859-0.899) for the negative subscale in this study.

Positive and negative emotions were assessed using the Chinese Affect Scale [48], which consists of 20 items with 2 subscales: positive affect (PA; 10 items) and negative affect (NA; 10 items). Participants rated the items on a 6-point Likert scale (1=“not at all” to 6=“extremely”). The scores for each subscale were summed, with higher scores indicating higher levels of PA and NA, respectively. The Cronbach α coefficients were 0.883 (95% CI 0.863-0.900) for the PA subscale and 0.876 (95% CI 0.853-0.894) for the NA subscale in this study.

### Statistical Analysis

All analyses followed the CONSORT (Consolidated Standards of Reporting Trials) statement for RCTs [49]. Assignment completion and dropout were analyzed using a chi-square test and logistic regression. Missing data were imputed with m=5 data sets using multiple imputations with chained equations and predictive mean matching [50]. Analyses were conducted according to the intent-to-treat approach and performed on imputed data sets before pooling the coefficients. Analyses were conducted within a linear mixed model framework [51]. Outcomes at baseline, age, gender, and medication use were included as covariates. Sensitivity analyses using completer-only data were also conducted. When comparing the active treatments (web-based iCBT vs app-based iCBT) at posttreatment, and at 3 and 6 months, models included treatment, time, and the interaction between time and treatment. The within-group effects of transitioning from WLC to active treatments were modeled in a separate analysis, using the second premeasurement as a baseline (see Table S4 in Multimedia Appendix 2). All models used restricted maximum likelihood estimation and an unstructured covariance matrix. Chi-square tests and logistic regression were conducted to explore differences in baseline characteristics between those who completed all modules and those who did not, as well as between those who completed postmeasurements and those who did not. Effect sizes were calculated using Cohen *d* [52] with the emmeans package [53], following the standard convention for magnitudes of effect sizes: small (*d*=0.2), medium (*d*=0.5), and large (*d*≥0.8). Data analyses were performed in R version 4.3.1 (R Foundation) [54].

Clinical significance was calculated using the Jacobson-Truax method [55]. “Recovered” was defined as a positive change that is both reliable (ie, not due to measurement error) and clinically significant (ie, an improvement of 2 SDs from the baseline sample mean); “improved” was defined as a reliable improvement, but not clinically significant; “unchanged” was defined as no reliable change; and “deteriorated” was defined as a reliable worsening. Following van Ballegooijen et al’s [26] suggestion, those who were given an intervention but did not commence treatment were also included in the calculation of the adherence rate. Adherence was analyzed separately for treatment modules and counseling sessions. Treatment adherence was defined as completing all 8 treatment modules, and counseling adherence was defined as participating in all 4 counseling sessions. The calculation was based on the data at the posttest.

## Results

### Participant Flow

**Figure 1:**
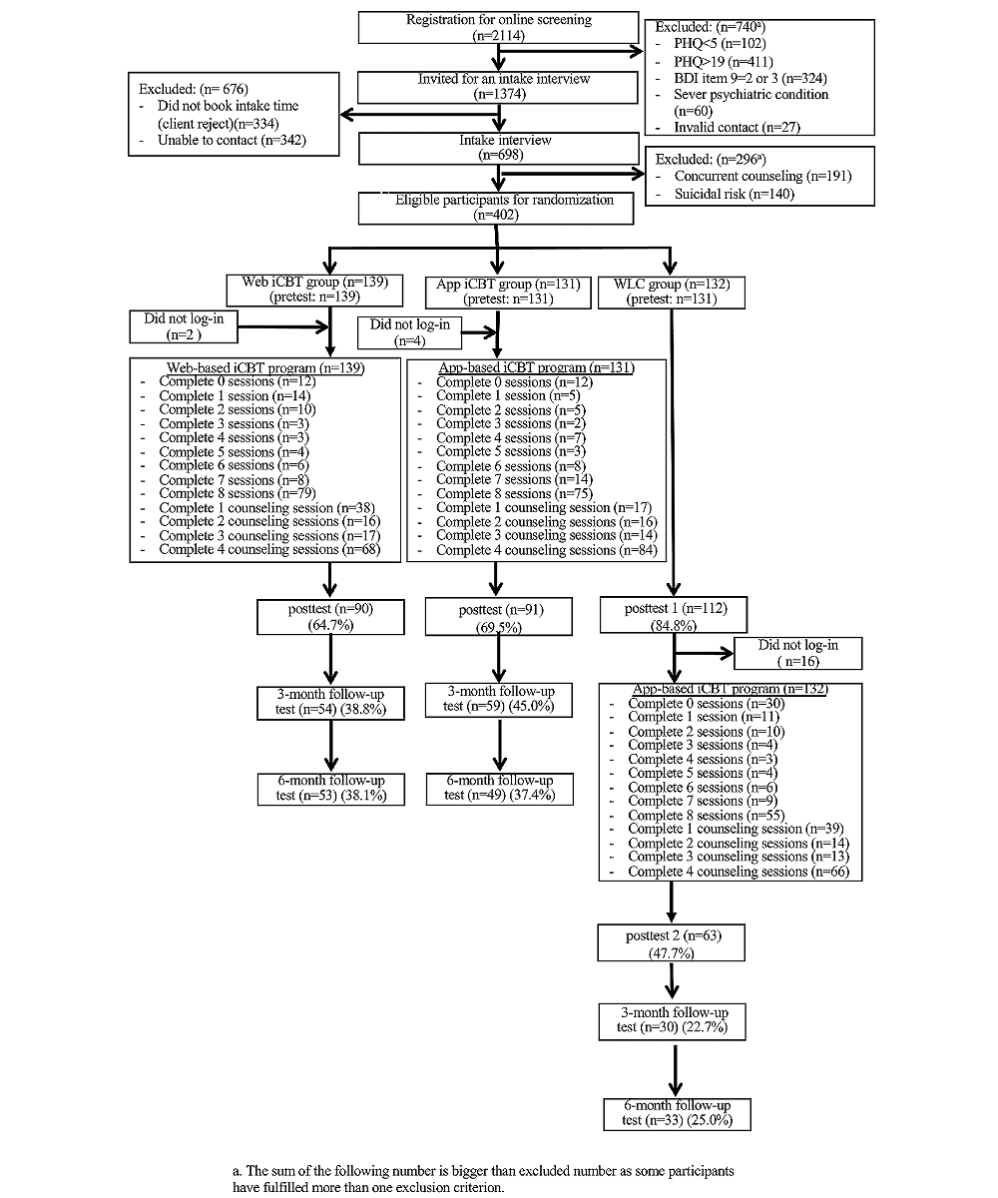
Participant flow of the study. iCBT: internet-based cognitive behavioral therapy.

A total of 2114 participants registered for the CANDO Program’s online screening, and 1374 were invited for an intake interview for further assessment. Of these, 698 intake interviews were conducted, and ultimately, 402 participants who met the inclusion criteria were randomly assigned to 1 of the 3 groups: web-based iCBT (n=139), app-based iCBT (n=131), and WLC (n=132). All allocated participants, except for 1 in the WLC group, completed a pretest questionnaire and were included in the intent-to-treat analysis; 2, 4, and 16 eligible participants did not log into the CANDO system after being assigned a client account in the web-based iCBT, app-based iCBT, and WLC groups, respectively, resulting in 137, 127, and 116 participants in the corresponding intervention conditions. At the posttest, 90, 91, and 112 participants provided data in the web-based iCBT, app-based iCBT, and WLC groups, respectively. A total of 116 participants from the WLC group later joined the web-based iCBT program, with 63, 30, and 33 participants providing data at posttreatment, 3-month, and 6-month follow-up tests, respectively. For the web-based/app-based iCBT groups, 54/59 and 53/49 participants provided data at the 3- and 6-month follow-up tests, respectively. See Figure 1 for the participant flowchart. Also see Multimedia Appendix 3.

### Participant Characteristics

Table 1 summarizes the participant data at baseline. Among the 402 participants who were randomized, 313 (77.9%) were female and 89 (22.1%) were male; 225 (56.0%) were single and 130 (32.3%) were married. The mean age was 37.3 (SD 11.0) years. The majority of participants (n=310, 77.1%) had university degrees, 85 (21.1%) and 1 (0.2%) had completed secondary and primary school education, respectively. A total of 77 (19.2%) participants were diagnosed with depression, 29 (7.2%) with anxiety, and 39 (9.7%) with other mental health conditions. Furthermore, 83 participants (20.6%) reported a history of self-harm, and 17 (4.2%) had previously attempted suicide; 99 participants (24.6%) reported using medication.

**Table 1:** Participant demographic characteristics.

Characteristics		Total (n=402)	Web group (n=139)	App group (n=131)	WLC^a^ group (n=132)
Age, mean (SD)		37.3 (11.0)	38.1 (11.0)	37.2 (11.5)	36.6 (10.6)
Female, n (%)		313 (77.9)	106 (76.3)	100 (76.3)	107 (81.1)
**Education, n (%)**					
	Primary school or below	1 (0.2)	1 (0.7)	0 (0.0)	0 (0.0)
	Secondary school	85 (21.1)	24 (17.3)	36 (27.5)	25 (18.9)
	University or above	310 (77.1)	111 (79.9)	94 (71.8)	105 (79.5)
	Missing	6 (1.5)	3 (2.2)	1 (0.8)	2 (1.5)
**Marital status, n (%)**					
	Single	225 (56.0)	78 (56.1)	72 (55.0)	75 (56.8)
	Married	130 (32.3)	43 (30.9)	44 (33.6)	43 (32.6)
	Other	30 (7.5)	10 (7.2)	11 (8.4)	9 (6.8)
	Missing	17 (4.2)	8 (5.8)	4 (3.1)	5 (3.8)
**Diagnosis, n (%)**					
	Depression	77 (19.2)	26 (18.7)	29 (22.1)	22 (16.7)
	Anxiety	29 (7.2)	12 (8.6)	8 (6.1)	9 (6.8)
	Other	39 (9.7)	16 (11.5)	14 (10.7)	9 (6.8)
	Missing	257 (63.9)	85 (61.2)	80 (61.1)	92 (69.7)

^a^WLC: waitlist control.

### Treatment Adherence and Attrition Rate

Following van Ballegooijen et al’s [26] suggestion, those who were given an intervention but did not commence treatment were also included in the calculation of the adherence rate. Thus, for adherence to the online modules, 79 of 139 (56.8%) participants in the web-based iCBT group and 75 of 131 (57.3%) in the app-based iCBT group completed all 8 modules, resulting in an overall adherence rate of over 50% (154/270, 57.0%). For the counseling sessions, 68 of 139 (48.9%) participants in the web-based group and 84 of 131 (64.1%) in the app-based group attended all 4 sessions (overall adherence rate: 152/270, 56.3%) The participants in the web- and app-based iCBT groups completed an average of 5.96 out of 8 modules (74.5%) and 5.77 out of 8 assignments (72.1%), respectively. In terms of module completion rate, there was no significant difference between the web-based (79/139, 56.8%) and app-based (75/131, 57.3%, *χ*^2^_1_=0.005, *P*=.95) groups. Similarly, no significant difference was found in the assignment completion rate between the web-based (70/139, 50.4%) and app-based (69/131, 52.7%, *χ*^2^_1_=0.07, *P*=.8) groups. Furthermore, there was no association between assignment completion and gender (*χ*^2^_1_=0.97, *P*=.32) or medication (*χ*^2^_1_=0.52, *P*=.47). However, a positive association was found between assignment completion and age (odds ratio 1.04, *P*=.003). Thus, older participants completed more assignments. Regarding the attrition rate for questionnaire submission, 89 out of 270 (33.0%), 157 out of 270 (58.1%), and 168 out of 270 (62.2%) participants in the 2 iCBT groups were lost to follow-up at posttest, 3-month, and 6-month follow-ups, respectively. The submission rates for the questionnaires are shown in Figure 1. Adherence to completing the postmeasurements was similar to module adherence, with older individuals being more likely to complete the postmeasurement (odds ratio 1.02, *P*=.047). However, there was no association between completing the postmeasurement and model covariates such as gender (*χ*^2^_1_=1.07, *P*=.30), treatment group (*χ*^2^_1_=0.48, *P*=.49), or medication (*χ*^2^_1_=0.003, *P*=.95). A chi-square test showed that there were no significant differences in demographic characteristics between completers and noncompleters of the postmeasurement and all 8 online modules (see Tables S2 and S3 in Multimedia Appendix 2).

### Treatment Effects at Posttest

**Figure 2:**
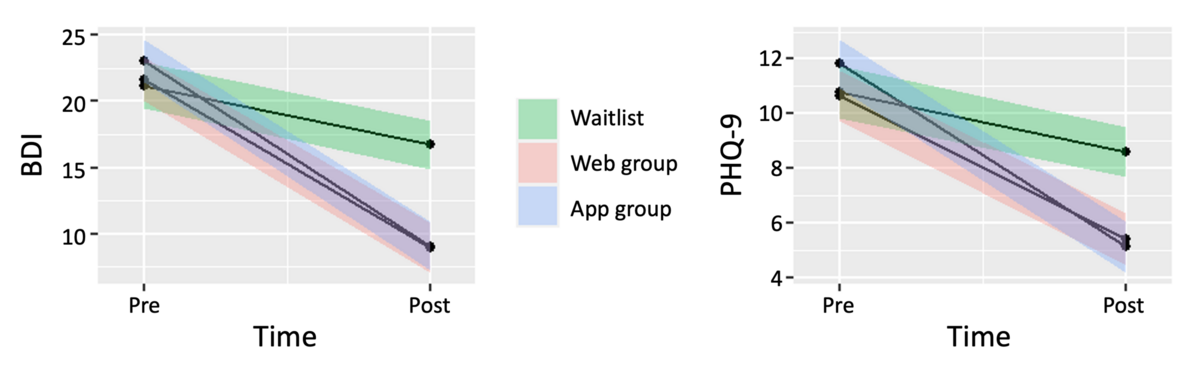
Treatment effects for primary outcomes BDI and PHQ-9. Shaded regions show 95% CIs. BDI: Beck Depression Inventory; PHQ-9: 9-item Patient Health Questionnaire.

The treatment effects at posttest, comparing the 2 iCBT groups with the WLC group, are shown in Table 2 and Figure 2. The primary analysis, which compared the 2 active treatments with the WLC at posttreatment using the data set with multiple imputed data, showed that both iCBT groups were superior to the WLC group. The adjusted differences on the BDI-II were –8.24 (95% CI –5.96 to –10.52; *P*<.001) for the web-based iCBT group, and –9.38 (95% CI –7.10 to –11.65; *P*<.001) for the app-based iCBT group. Likewise, the treatment effects for the PHQ-9 were also superior to the WLC group, with an adjusted score of –3.07 (95% CI –1.67 to –4.46; *P*<.001) for the web-based iCBT group and –4.50 (95% CI –3.12 to –5.89; *P*<.001) for the app-based iCBT group. All secondary outcome measures showed significant improvement compared with the WLC at postmeasurement (*P*=.009). The between-group effect sizes for the BDI-II were *d*=1.07 (95% CI 0.81-1.34) and *d*=1.15 (95% CI 0.88-1.43) for the web-based and app-based iCBT groups, respectively. For the PHQ-9, the corresponding between-group effect sizes were *d*=0.78 (95% CI 0.52-1.04) and *d*=0.95 (95% CI 0.63-1.27) for the web-based and app-based iCBT groups, respectively. Sensitivity analyses using the completer-only data set yielded similar results (see Table S1 in Multimedia Appendix 2). The within-group effect sizes for the BDI-II at posttest were *d*=1.49 (95% CI 1.19-1.78) for the web-based iCBT group and *d*=1.64 (95% CI 1.33-1.95) for the app-based iCBT group. The corresponding effect sizes for the PHQ-9 at posttest were *d*=1.11 (95% CI 0.81-1.41) for the web-based iCBT group and *d*=1.70 (95% CI 1.38-2.02) for the app-based iCBT group. The WLC group showed significant improvement on all outcome measures, with medium to large within-group effect sizes when transitioning to the web-based iCBT (see Table S4 in Multimedia Appendix 2).

**Table 2:** Comparison of treatment effects between active and WLC^a^ groups at post-treatment (intent-to-treat analysis).

Outcome	Unadjusted mean (SD)			Adjusted difference (95% CI)				Between group effect size *d* (95% CI)		Within group effect size (95% CI)		
	Web	App	WL	Web vs WL	*P* value	App vs WL	*P* value	Web vs WL	App vs WL	Web	App	WLC
BDI-II^b^	9.40 (8.90)	9.43 (8.45)	18.02 (9.02)	–8.24 (–10.52 to –5.96)	<.001	–9.38 (–11.65 to –7.10)	<.001	1.07 (0.81 to 1.34)	1.15 (0.88 to 1.43)	1.48 (1.18 to 1.78)	1.64 (1.33 to 1.94)	0.45 (0.2 to 0.71)
PHQ-9^c^	5.59 (4.43)	5.24 (3.65)	8.99 (4.58)	–3.07 (–4.46 to –1.67)	<.001	–4.50 (–5.89 to –3.12)	<.001	0.78 (0.52 to 1.04)	0.95 (0.63 to 1.27)	1.11 (0.81 to 1.41)	1.69 (1.36 to 2.02)	0.46 (0.19 to 0.73)
*GHQ-12^d^*	1.60 (3.06)	1.53 (2.77)	5.35 (3.85)	–3.65 (–4.75 to –2.54)	<.001	–4.49 (–5.6 to –3.38)	<.001	1.05 (0.77 to 1.32)	1.11 (0.83 to 1.39)	1.64 (1.34 to 1.95)	2.21 (1.87 to 2.55)	0.64 (0.38 to 0.9)
*BAI^e^*	8.89 (7.89)	8.49 (6.69)	11.09 (7.49)	–2.47 (–4.33 to –0.62)	.009	–3.32 (–5.17 to –1.47)	<.001	0.37 (0.11 to 0.63)	0.54 (0.29 to 0.8)	0.64 (0.37 to 0.91)	0.88 (0.59 to 1.15)	0.37 (0.11 to 0.62)
Positive automatic thoughts	2.40 (0.71)	2.33 (0.67)	1.98 (0.58)	0.38 (0.22 to 0.54)	<.001	0.40 (0.24 to 0.55)	<.001	–0.63 (–0.9 to –0.37)	–0.61 (–0.9 to –0.32)	–0.88 (–1.16 to –0.6)	–0.93 (–1.22 to –0.65)	–0.30 (–0.55 to 0.04)
Negative automatic thoughts	1.85 (0.78)	1.89 (0.63)	2.29 (0.80)	–0.43 (–0.63 to –0.22)	<.001	–0.48 (–0.68 to –0.27)	<.001	0.63 (0.37 to 0.90)	0.66 (0.35 to 0.97)	0.83 (0.55 to 1.11)	1.00 (0.72 to 1.28)	0.28 (0.02 to 0.53)
Positive emotions	3.26 (0.69)	3.27 (0.62)	2.89 (0.68)	0.35 (0.18 to 0.52)	<.001	0.43 (0.26 to 0.60)	<.001	–0.56 (–0.84 to –0.27)	–0.60 (–0.9 to –0.3)	–0.89 (–1.17 to –0.61)	–1.10 (–1.38 to –0.81)	–0.30 (–0.55 to –0.04)
Negative emotions	2.91 (0.82)	2.92 (0.69)	3.33 (0.74)	–0.45 (–0.64 to –0.25)	<.001	–0.47 (–0.66 to –0.27)	<.001	0.64 (0.37 to 0.91)	0.65 (0.38 to 0.91)	1.06 (0.78 to 1.34)	1.21 (0.91 to 1.5)	0.50 (0.24 to 0.75)

^a^WLC: waitlist control.

^b^BDI-II: Beck Depression Inventory-II.

^c^PHQ-9: 9-item Patient Health Questionnaire.

^d^GHQ-12: 12-item General Health Questionnaire.

^e^BAI: Beck Anxiety Inventory.

### Reliable Changes at Postintervention Assessments

The frequencies and percentages of reliable change are shown in Table 3. At posttest, using the BDI-II as the outcome measure, reliable recovery was observed in 35 out of 90 (38.9%) participants in the web-based iCBT group, 36 out of 91 (39.6%) in the app-based iCBT group, and 3 out of 112 (2.7%) in the WLC group. For the PHQ-9 at posttest, the reliable recovery rates were 16 out of 67 (24%) for the web-based iCBT group, 16 out of 72 (22%) for the app-based iCBT group, and 5 out of 88 (6%) for the WLC group. Chi-square tests of association revealed a significant difference in the distribution of the 3 groups for both the BDI-II (*χ*^2^_6_=71.92, *P*<.001) and the PHQ-9 (*χ*^2^_6_=33.78, *P*<.001). When comparing clinical significance between the 2 treatment groups, no significant differences were found (*χ*^2^_3_=3.24, *P*=.35).

The reliable recovery rates for BDI-II at the 6-month follow-up were 13 out of 53 (25%) for the web-based iCBT group and 14 out of 49 (29%) for the app-based iCBT group. For PHQ-9 at the 6-month follow-up, the corresponding reliable recovery rates were 4 out of 40 (10%) for the web-based iCBT group and 3 out of 38 (8%) for the app-based iCBT group.

**Table 3:** Reliable and clinically significant changes summarized over group and time.

Outcome			Web group, n/N (%)		App group, n/N (%)		WLC^a^ group, n/N (%)		Pearson chi-square (*df*)		*P* value
**BDI-II^b^, posttest**									71.92 (6)		<.001
	Recovered	35/90 (38.9)		36/91 (39.6)		3/110 (2.7)		N/A^c^		N/A	
	Improved	28/90 (31.1)		36/91 (39.6)		31/110 (28.2)		N/A		N/A	
	Unchanged	27/90 (30.0)		17/91 (18.7)		70/110 (63.6)		N/A		N/A	
	Deteriorated	0/90 (0)		2/91 (2.2)		6/110 (5.5)		N/A		N/A	
**PHQ-9^d^, posttest**									33.78 (6)		<.001
	Recovered	16/67 (23.9)		16/72 (22.2)		5/88 (5.7)		N/A		N/A	
	Improved	22/67 (32.8)		39/72 (54.2)		26/88 (29.5)		N/A		N/A	
	Unchanged	28/67 (41.8)		15/72 (20.8)		50/88 (56.8)		N/A		N/A	
	Deteriorated	1/67 (1.5)		2/72 (2.8)		7/88 (8)		N/A		N/A	
**BDI-II, 3-month follow-up**									3.24 (3)		.35
	Recovered	21/54 (38.9)		20/59 (33.9)		N/A		N/A		N/A	
	Improved	20/54 (37.0)		27/59 (45.8)		N/A		N/A		N/A	
	Unchanged	13/54 (24.1)		10/59 (16.9)		N/A		N/A		N/A	
	Deteriorated	0/54 (0)		2/59 (3.4)		N/A		N/A		N/A	
**PHQ-9, 3-month follow-up**									5.39 (3)		0.14
	Recovered	8/46 (17.4)		7/46 (15.2)		N/A		N/A		N/A	
	Improved	20/46 (43.5)		27/46 (58.7)		N/A		N/A		N/A	
	Unchanged	18/46 (39.1)		10/46 (21.7)		N/A		N/A		N/A	
	Deteriorated	0/46 (0)		2/46 (4.3)		N/A		N/A		N/A	
**BDI-II, 6-month follow-up**									6.32 (3)		.10
	Recovered	13/53 (24.5)		14/49 (28.6)		N/A		N/A		N/A	
	Improved	22/53 (41.5)		17/49 (34.7)		N/A		N/A		N/A	
	Unchanged	17/53 (32.1)		11/49 (22.4)		N/A		N/A		N/A	
	Deteriorated	1/53 (1.9)		7/49 (14.3)		N/A		N/A		N/A	
**PHQ-9, 6-month follow-up**									2.75 (3)		.43
	Recovered	4/40 (10.0)		3/38 (7.9)		N/A		N/A		N/A	
	Improved	14/40 (35.0)		18/38 (47.4)		N/A		N/A		N/A	
	Unchanged	20/40 (50.0)		13/38 (34.2)		N/A		N/A		N/A	
	Deteriorated	2/40 (5.0)		4/38 (10.5)		N/A		N/A		N/A	

^a^WLC: waitlist control.

^b^BDI-II: Beck Depression Inventory-II.

^c^N/A: not applicable.

^d^PHQ-9: 9-item Patient Health Questionnaire.

### Comparisons Between Two iCBT Groups at Posttreatment Assessments

At posttreatment, the web-based iCBT group did not show a significant difference in BDI-II (adjusted difference 0.21, 95% CI –2.28 to 2.70, *P*=.87) or PHQ-9 (adjusted difference 0.35, 95% CI –1.08 to 1.77, *P*=.63) scores compared with the app-based iCBT group. Similarly, at the 3- and 6-month follow-ups, the differences between the 2 iCBT groups in their BDI-II and PHQ-9 scores remained nonsignificant (Table 4). For the secondary outcome measures, no significant differences were found between the 2 groups on any outcome measure at any time point, except for the GHQ-12 at 6 months, which favored the web group (adjusted difference –1.46, 95% CI –0.33 to –2.59, *P*=.01). Compared with the baseline, the within-group effect sizes ranged from 0.54 to 2.21 at posttreatment assessments for both the web- and app-based iCBT groups (Table 4). Thus, both iCBT groups maintained treatment effects at the 3- and 6-month follow-ups for most of the outcome measures.

**Table 4:** Comparisons between web-based and app-based iCBT^a^ groups on primary and secondary outcome measures at post-treatment (intention-to-treat analysis).

Measure and time		Unadjusted mean (SD)		Adjusted difference (95% CI)	*P* value	Between-group effect size *d* (95% CI)	Within-group effect size (95% CI)	
		Web	App				Web	App
**BDI-II^b^**								
	Baseline	22.24 (8.52)	23.66 (8.77)	N/A^c^	N/A	N/A	N/A	N/A
	Post	9.40 (8.90)	9.*43* (8.4*5*)	0.21 (–2.28 to 2.70)	.87	–0.01 (–0.3 to 0.28)	1.48 (1.18 to 1.78)	1.64 (1.33 to 1.94)
	3 months	9.43 (9.58)	9.15 (8.51)	–0.14 (–3.11 to 2.83)	.93	0.03 (–0.34 to 0.4)	1.45 (1.1 to 1.8)	1.67 (1.32 to 2.02)
	6 months	10.15 (9.03)	13.47 (12.24)	–1.37 (–4.46 to 1.72)	.38	–0.37 (–0.76 to 0.02)	1.40 (1.05 to 1.74)	1.04 (0.69 to 1.38)
**PHQ-9^d^**								
	Baseline	10.77 (4.9)	12.07 (4.27)	N/A	N/A	N/A	N/A	N/A
	Post	5.59 (4.43)	5.2*4* (3.6*5*)	0.35 (–1.08 to 1.77)	.63	0.07 (–0.22 to 0.36)	1.11 (0.8 to 1.41)	1.69 (1.36 to 2.02)
	3 months	5.41 (4.01)	5.83 (3.57)	–0.27 (–1.94 to 1.41)	.75	–0.1 (–0.47 to 0.28)	1.16 (0.81 to 1.51)	1.55 (1.18 to 1.91)
	6 months	5.94 (5.44)	7.65 (5.06)	–0.86 (–2.67 to 0.94)	.35	–0.39 (–0.79 to 0.01)	0.95 (0.59 to 1.31)	0.97 (0.62 to 1.33)
**GHQ-12^e^**								
	Baseline	7.46 (3.86)	8.4 (3.31)	N/A	N/A	N/A	N/A	N/A
	Post	1.60 (3.06)	1.53 (2.7*7*)	0.05 (–0.84 to 0.93)	.91	0.02 (–0.27 to 0.31)	1.64 (1.34 to 1.95)	2.21 (1.87 to 2.55)
	3 months	1.85 (3.02)	1.69 (2.82)	–0.08 (–1.17 to 1)	.88	0.05 (–0.32 to 0.42)	1.54 (1.19 to 1.89)	2.12 (1.74 to 2.49)
	6 months	1.77 (3.20)	3.49 (4.10)	–1.46 (–0.33 to –2.59)	.01	–0.52 (–0.91 to –0.12)	1.54 (1.19 to 1.89)	1.39 (1.03 to 1.74)
**BAI^f^**								
	Baseline	14.53 (9.29)	15.36 (8.8)	N/A	N/A	N/A	N/A	N/A
	Post	8.89 (7.89)	8.*49* (6.6*9*)	0.53 (–1.25 to 2.31)	.56	0.06 (–0.23 to 0.36)	0.64 (0.37 to 0.91)	0.88 (0.59 to 1.15)
	3 months	7.67 (6.42)	8.03 (6.78)	0.59 (–1.56 to 2.73)	.59	–0.05 (–0.42 to 0.32)	0.80 (0.47 to 1.12)	0.89 (0.57 to 1.21)
	6 months	7.89 (7.99)	9.88 (7.72)	0.32 (–1.91 to 2.56)	.78	–0.25 (–0.64 to 0.14)	0.74 (0.42 to 1.07)	0.64 (0.31 to 0.98)
**Positive automatic thoughts**								
	Baseline	1.85 (0.55)	1.79 (0.52)	N/A	N/A	N/A	N/A	N/A
	Post	2.4*0* (0.71)	2.33 (0.67)	0.01 (–0.18 to 0.2)	.93	0.1 (–0.19 to 0.39)	–0.88 (–1.16 to –0.6)	–0.93 (–1.21 to –0.65)
	3 months	2.49 (0.67)	2.44 (0.84)	–0.1 (–0.32 to 0.12)	.38	0.07 (–0.3 to 0.44)	–1.08 (–1.41 to –0.75)	–1.03 (–1.36 to –0.71)
	6 months	2.46 (0.85)	2.15 (0.71)	0.08 (–0.16 to 0.31)	.52	0.47 (0.08 to 0.86)	–0.93 (–1.26 to –0.6)	–0.63 (–0.96 to –0.29)
**Negative automatic thoughts**								
	Baseline	2.55 (0.88)	2.65 (0.85)	N/A	N/A	N/A	N/A	N/A
	Post	1.85 (0.78)	1.8*9* (0.6*3*)	–0.02 (–0.22 to 0.18)	.86	–0.04 (–0.33 to 0.25)	0.83 (0.55 to 1.1)	1 (0.72 to 1.28)
	3 months	1.74 (0.68)	1.82 (0.71)	–0.03 (–0.27 to 0.21)	.81	–0.09 (–0.46 to 0.28)	0.97 (0.64 to 1.29)	1.02 (0.7 to 1.34)
	6 months	1.75 (0.78)	2.05 (0.93)	–0.15 (–0.4 to 0.1)	.24	–0.38 (–0.77 to 0.01)	0.93 (0.6 to 1.26)	0.68 (0.34 to 1.02)
**Positive emotions**								
	Baseline	2.72 (0.54)	2.65 (0.54)	N/A	N/A	N/A	N/A	N/A
	Post	3.26 (0.69)	3.2*7* (0.62)	–0.03 (–0.22 to 0.16)	.87	–0.04 (–0.33 to 0.25)	–0.89 (–1.17 to –0.61)	–1.1 (–1.38 to –0.81)
	3 months	3.26 (0.75)	3.33 (0.81)	–0.1 (–0.33 to 0.13)	.93	–0.12 (–0.49 to 0.25)	–0.89 (–1.21 to –0.56)	–1.07 (–1.39 to –0.74)
	6 months	3.24 (0.75)	2.97 (0.72)	0.12 (–0.11 to 0.36)	.38	0.4 (0.01 to 0.79)	–0.85 (–1.18 to –0.52)	–0.54 (–0.88 to –0.21)
**Negative emotions**								
	Baseline	3.73 (0.75)	3.78 (0.73)	N/A	N/A	N/A	N/A	N/A
	Post	2.91 (0.82)	2.9*3* (0.*69*)	–0.02 (–0.23 to 0.2)	.63	–0.02 (–0.31 to 0.28)	1.06 (0.78 to 1.34)	1.21 (0.91 to 1.5)
	3 months	2.78 (0.85)	2.93 (0.73)	–0.07 (–0.32 to 0.18)	.75	–0.19 (–0.56 to 0.18)	1.23 (0.89 to 1.57)	1.17 (0.84 to 1.5)
	6 months	2.86 (0.91)	3.17 (0.88)	–0.1 (–0.36 to 0.16)	.35	–0.39 (–0.78 to 0)	1.09 (0.75 to 1.42)	0.8 (0.46 to 1.13)

^a^iCBT: internet-based cognitive behavioral therapy

^b^BDI-II: Beck Depression Inventory-II.

^c^N/A: not applicable.

^d^PHQ-9: 9-item Patient Health Questionnaire.

^e^GHQ-12: 12-item General Health Questionnaire.

^f^BAI: Beck Anxiety Inventory.

## Discussion

### Effectiveness of iCBT for Hong Kong Chinese

To the best of our knowledge, this study is one of the few to develop and test the effectiveness of iCBT among Hong Kong Chinese. Both the web- and app-based iCBT groups were superior to the WLC in managing depressive symptoms. The findings suggest that a culturally and linguistically appropriate, therapist-guided iCBT program yields positive therapeutic effects, with a good recovery rate and treatment adherence, for Hong Kong Chinese with mild to moderate depressive symptoms. These positive effects are maintained at the 3- and 6-month follow-ups, including reductions in depression and anxiety symptoms, psychological distress, negative automatic thoughts, and negative emotions, as well as increases in positive automatic thoughts and emotions. The 2 delivery modalities of iCBT showed similar intervention effects on most of the outcome measures at posttreatment and follow-up tests. These findings are consistent with those reported in Western contexts [10], China (eg, [16,18]), and Hong Kong [19,20]. The recovery rates at posttreatment, as measured by the BDI-II, were 39% (35/90) and 40% (36/91) for web- and app-based iCBT, respectively. These rates align with those (30%-55%) reported in RCT studies involving Western participants [24,25], but are lower than the 58% recovery rate reported in an RCT study of iCBT conducted in hospitals with Chinese patients with depression in China [16]. The positive effects of iCBT on depression treatment are comparable to face-to-face CBT in terms of symptom reduction (eg, [56]) and recovery rate (eg, [57]), suggesting that iCBT does not compromise treatment outcomes.

Like CBT, it appears that a culturally adapted iCBT works well for Chinese people. The positive effects of the CANDO Program may be attributed to the cultural and linguistic adaptations of iCBT for Hong Kong Chinese. First, the program is delivered in Cantonese, the mother tongue of Hong Kong residents, to facilitate their understanding of the content. Additionally, 4-character Chinese idioms [31] are used to describe cognitive distortions, ensuring an accurate understanding of the CBT concepts and skills. Second, many real-life examples are incorporated into the case videos to demonstrate the application of CBT skills in coping with depression in the context of Hong Kong. These examples may resonate with participants’ own automatic thoughts and cognitive rules, enhancing their self-learning of CBT skills. Third, laypersons—such as family members, friends, and colleagues—who have experience and knowledge of CBT appear in the case demonstration videos (except for 1 case) as therapists. This approach helps normalize the help-seeking and counseling process, reducing the stigma surrounding mental health issues and making it more acceptable to Hong Kong Chinese. Fourth, the CANDO Program is video-based with limited text narrative, which aligns with the preferred approach of Hong Kong people for receiving new information and learning new knowledge and skills through watching videos. Fifth, the program includes relaxation exercises to enhance the Chinese philosophy of body-mind connection, which complements the CBT framework that links the body, cognition, and emotions. Finally, the therapist is a Chinese clinical psychologist with training and practical experience in both Hong Kong and the United States, ensuring cultural sensitivity in working with Hong Kong Chinese.

### Program Adherence and Attrition

The percentage of completed sessions in this study (5.96/8, 75%) is slightly lower than those reported in meta-analyses (80.8% in [26] and 76.0% in [10]), but much higher than that reported in a Hong Kong study (52.7% in [21]) and lower than that in a study in China (83.8% in [18]). The percentage of participants who completed all the sessions in this study (154/270, 57.0%) is similar to those reported in [26] (65.1%) and other empirical studies (eg, 60% in [28] and 67% in [21]), but lower than that in [18] (81.4%). Overall, the program adherence in this study is comparable to that in previous studies, though slightly lower than that in [18]. One reason for this difference may be related to the number of sessions (8 sessions in the CANDO Program compared with 5 in [18]), as it is generally easier to complete an iCBT program with fewer sessions. Another factor is the setting; Ying et al [18] conducted their study in a hospital, where doctors acted as therapists and provided relatively intensive guidance, which likely encouraged greater program engagement. The Chinese cultural tendency to adhere to the authority of medical professionals may have contributed to the higher adherence observed in their study.

Consistent with previous findings, this study also observed a high attrition rate at postintervention. Specifically, the attrition rate at posttest (89/270, 33.0%) is comparable to the average dropout rate of 28% found in therapist-guided computer-based intervention studies in [58], which is higher than the approximately 17% dropout rate in RCT studies of face-to-face CBT [58]. The high attrition rates in this study are also consistent with those reported in Hong Kong studies [21,59]. One possible reason for the high attrition rate may be the fast-paced lifestyle of Hong Kong residents, which limits their ability to commit to the program. Additionally, a lack of motivation to participate could contribute to the dropout rate. Similar to [21], the participants in this study did not have a formal diagnosis of a mental disorder and did not have an immediate need for help-seeking or intervention retention.

No significant difference in program effectiveness or adherence was found between the web- and app-based iCBT groups among Hong Kong people with mild to moderate levels of depression. This suggests that the medium of service delivery does not impact the intervention effects, with both formats being equally effective.

### Theoretical and Practice Implications

At the theoretical level, the findings offer empirical evidence supporting the effectiveness of iCBT for handling depressive symptoms in Chinese communities. Culturally and linguistically adapted iCBT ensures its relevance and effectiveness for the Chinese population, particularly for Hong Kong Chinese. The use of the internet and mobile app for delivering CBT represents a significant advancement in mental health care, especially in Hong Kong, where the high penetration of digital devices facilitates the widespread adoption of iCBT. This study provides a strong evidence base that supports the adoption and further development of iCBT in Hong Kong. At the practice level, this study demonstrates that Hong Kong Chinese are receptive to iCBT, making it a viable alternative that can be integrated into traditional mental health services. Furthermore, iCBT is cost-effective. This study, conducted during the COVID-19 pandemic when most face-to-face mental health services were suspended, highlights the significant advantage of iCBT in terms of the therapist-to-client ratio. For the CANDO Program, the therapist-to-client ratio is 1:190 per year, which is much higher than the traditional face-to-face therapy ratio of 1:40-50 in Hong Kong [60]. This is especially relevant to Hong Kong, where there is a severe shortage of mental health professionals and long wait times for traditional services. iCBT offers a scalable and viable solution to address this service gap in the long term. Therefore, more resources, particularly from the government, should be allocated to developing additional iCBT programs for mental health conditions, training more iCBT therapists, and integrating iCBT into the infrastructure of existing mental health care systems in Hong Kong.

### Limitations

One major limitation of this study is the high attrition rate in questionnaire submission postintervention, despite various efforts to encourage participation. Although this is common, it may introduce nonresponse bias. Future studies should aim to reduce the attrition rate through improvements in program design and service delivery. A second limitation is that the therapist’s time per client was not recorded, making it difficult to evaluate the cost-effectiveness of the program more accurately. Future studies should track these data to better assess the program’s cost-effectiveness. Third, client satisfaction data were not collected, which could provide valuable insights into user experience and should be considered in future research. Finally, a large proportion of participants were female (313/402, 77.9%) and highly educated (310/402, 77.1%, holding a university degree or higher), which may limit the generalizability of the findings to the broader Hong Kong population. While gender was controlled as a covariate in the data analysis, future studies should aim to recruit more male participants and those with lower levels of education to improve representativeness.

### Conclusions

This RCT study demonstrates that both web- and app-based iCBT are effective in reducing depression and anxiety symptoms, psychological distress, and negative automatic thoughts and emotions, while enhancing positive automatic thoughts and emotions in Hong Kong Chinese with mild to moderate depressive symptoms. The study also shows good program adherence and recovery rates, although the attrition rate for questionnaire submission postintervention was relatively low. ICBT is a promising approach in Hong Kong and warrants increased resource allocation for further development and implementation.

## Data Availability

The data sets generated and analyzed during this study are not publicly available due to privacy concerns but can be obtained from the corresponding author upon reasonable request.

## Appendix

Multimedia Appendix 1 Some screenshots of the Confront and Navigate Depression Online (CANDO) Program.

Multimedia Appendix 2 Additional data analysis.

Multimedia Appendix 3 CONSORT-eHEALTH checklist (V 1.6.1).

## Abbreviations

BAI: Beck Anxiety Inventory
BDI-II: Beck Depression Inventory-II
CANDO: Confront and Navigate Depression Online
CBT: cognitive behavioral therapy
CONSORT: Consolidated Standards of Reporting Trials
GHQ-12: 12-item General Health Questionnaire
iCBT: internet-based cognitive behavioral therapy
NA: negative affect
PA: positive affect
PHQ-9: 9-item Patient Health Questionnaire
RCT: randomized controlled trial
WHO: World Health Organization
WLC: waitlist control
