# Correction: Culturally Adapted Guided Internet-Based Cognitive Behavioral Therapy for Hong Kong People With Depressive Symptoms: Randomized Controlled Trial

**DOI:** 10.2196/88495

**Published:** 2026-02-03

**Authors:** Jia-Yan Pan, Jonas Rafi

**Affiliations:** 1 Department of Social Work Hong Kong Baptist University Hong Kong China (Hong Kong); 2 Department of Psychology Stockholm University Stockholm Sweden

In “Culturally Adapted Guided Internet-Based Cognitive Behavioral Therapy for Hong Kong People With Depressive Symptoms: Randomized Controlled Trial”[1], the authors noted some errors.

One sentence has been changed. In the “Measurements” section, the third sentence in the third paragraph originally appeared as follows:

Item scores were summed for the positive and negative subscales, with higher total scores indicating more positive and negative automatic thoughts, respectively.

This sentence now reads:

Item scores were averaged for the positive and negative subscales, with higher total scores indicating more positive and negative automatic thoughts, respectively.

The originally published Tables 2 and 4 have been revised due to typos. The new Table 2 and Table 4 that are replacing the original published versions are shown below (with changes marked in italics).

**Table 2:** Comparison of treatment effects between active and WLC^a^ groups at post-treatment (intent-to-treat analysis).

Outcome	Unadjusted mean (SD)			Adjusted difference (95% CI)				Between group effect size *d* (95% CI)		Within group effect size (95% CI)		
	Web	App	WL	Web vs WL	*P* value	App vs WL	*P* value	Web vs WL	App vs WL	Web	App	WLC
BDI-II^b^	9.40 (8.90)	9.43 (8.45)	18.02 (9.02)	–8.24 (–10.52 to –5.96)	<.001	–9.38 (–11.65 to –7.10)	<.001	1.07 (0.81 to 1.34)	1.15 (0.88 to 1.43)	1.48 (1.18 to 1.78)	1.64 (1.33 to 1.94)	0.45 (0.2 to 0.71)
PHQ-9^c^	5.59 (4.43)	5.24 (3.65)	8.99 (4.58)	–3.07 (–4.46 to –1.67)	<.001	–4.50 (–5.89 to –3.12)	<.001	0.78 (0.52 to 1.04)	0.95 (0.63 to 1.27)	1.11 (0.81 to 1.41)	1.69 (1.36 to 2.02)	0.46 (0.19 to 0.73)
*GHQ-12^d^*	1.60 (3.06)	1.53 (2.77)	5.35 (3.85)	–3.65 (–4.75 to –2.54)	<.001	–4.49 (–5.6 to –3.38)	<.001	1.05 (0.77 to 1.32)	1.11 (0.83 to 1.39)	1.64 (1.34 to 1.95)	2.21 (1.87 to 2.55)	0.64 (0.38 to 0.9)
*BAI^e^*	8.89 (7.89)	8.49 (6.69)	11.09 (7.49)	–2.47 (–4.33 to –0.62)	.009	–3.32 (–5.17 to –1.47)	<.001	0.37 (0.11 to 0.63)	0.54 (0.29 to 0.8)	0.64 (0.37 to 0.91)	0.88 (0.59 to 1.15)	0.37 (0.11 to 0.62)
Positive automatic thoughts	2.40 (0.71)	2.33 (0.67)	1.98 (0.58)	0.38 (0.22 to 0.54)	<.001	0.40 (0.24 to 0.55)	<.001	–0.63 (–0.9 to –0.37)	–0.61 (–0.9 to –0.32)	–0.88 (–1.16 to –0.6)	–0.93 (–1.22 to –0.65)	–0.30 (–0.55 to 0.04)
Negative automatic thoughts	1.85 (0.78)	1.89 (0.63)	2.29 (0.80)	–0.43 (–0.63 to –0.22)	<.001	–0.48 (–0.68 to –0.27)	<.001	0.63 (0.37 to 0.90)	0.66 (0.35 to 0.97)	0.83 (0.55 to 1.11)	1.00 (0.72 to 1.28)	0.28 (0.02 to 0.53)
Positive emotions	3.26 (0.69)	3.27 (0.62)	2.89 (0.68)	0.35 (0.18 to 0.52)	<.001	0.43 (0.26 to 0.60)	<.001	–0.56 (–0.84 to –0.27)	–0.60 (–0.9 to –0.3)	–0.89 (–1.17 to –0.61)	–1.10 (–1.38 to –0.81)	–0.30 (–0.55 to –0.04)
Negative emotions	2.91 (0.82)	2.92 (0.69)	3.33 (0.74)	–0.45 (–0.64 to –0.25)	<.001	–0.47 (–0.66 to –0.27)	<.001	0.64 (0.37 to 0.91)	0.65 (0.38 to 0.91)	1.06 (0.78 to 1.34)	1.21 (0.91 to 1.5)	0.50 (0.24 to 0.75)

^a^WLC: waitlist control.

^b^BDI-II: Beck Depression Inventory-II.

^c^PHQ-9: 9-item Patient Health Questionnaire.

^d^GHQ-12: 12-item General Health Questionnaire.

^e^BAI: Beck Anxiety Inventory.

**Table 4:** Comparisons between web-based and app-based iCBT^a^ groups on primary and secondary outcome measures at post-treatment (intention-to-treat analysis).

Measure and time		Unadjusted mean (SD)		Adjusted difference (95% CI)	*P* value	Between-group effect size *d* (95% CI)	Within-group effect size (95% CI)	
		Web	App				Web	App
**BDI-II^b^**								
	Baseline	22.24 (8.52)	23.66 (8.77)	N/A^c^	N/A	N/A	N/A	N/A
	Post	9.40 (8.90)	9.*43* (8.4*5*)	0.21 (–2.28 to 2.70)	.87	–0.01 (–0.3 to 0.28)	1.48 (1.18 to 1.78)	1.64 (1.33 to 1.94)
	3 months	9.43 (9.58)	9.15 (8.51)	–0.14 (–3.11 to 2.83)	.93	0.03 (–0.34 to 0.4)	1.45 (1.1 to 1.8)	1.67 (1.32 to 2.02)
	6 months	10.15 (9.03)	13.47 (12.24)	–1.37 (–4.46 to 1.72)	.38	–0.37 (–0.76 to 0.02)	1.40 (1.05 to 1.74)	1.04 (0.69 to 1.38)
**PHQ-9^d^**								
	Baseline	10.77 (4.9)	12.07 (4.27)	N/A	N/A	N/A	N/A	N/A
	Post	5.59 (4.43)	5.2*4* (3.6*5*)	0.35 (–1.08 to 1.77)	.63	0.07 (–0.22 to 0.36)	1.11 (0.8 to 1.41)	1.69 (1.36 to 2.02)
	3 months	5.41 (4.01)	5.83 (3.57)	–0.27 (–1.94 to 1.41)	.75	–0.1 (–0.47 to 0.28)	1.16 (0.81 to 1.51)	1.55 (1.18 to 1.91)
	6 months	5.94 (5.44)	7.65 (5.06)	–0.86 (–2.67 to 0.94)	.35	–0.39 (–0.79 to 0.01)	0.95 (0.59 to 1.31)	0.97 (0.62 to 1.33)
**GHQ-12^e^**								
	Baseline	7.46 (3.86)	8.4 (3.31)	N/A	N/A	N/A	N/A	N/A
	Post	1.60 (3.06)	1.53 (2.7*7*)	0.05 (–0.84 to 0.93)	.91	0.02 (–0.27 to 0.31)	1.64 (1.34 to 1.95)	2.21 (1.87 to 2.55)
	3 months	1.85 (3.02)	1.69 (2.82)	–0.08 (–1.17 to 1)	.88	0.05 (–0.32 to 0.42)	1.54 (1.19 to 1.89)	2.12 (1.74 to 2.49)
	6 months	1.77 (3.20)	3.49 (4.10)	–1.46 (–0.33 to –2.59)	.01	–0.52 (–0.91 to –0.12)	1.54 (1.19 to 1.89)	1.39 (1.03 to 1.74)
**BAI^f^**								
	Baseline	14.53 (9.29)	15.36 (8.8)	N/A	N/A	N/A	N/A	N/A
	Post	8.89 (7.89)	8.*49* (6.6*9*)	0.53 (–1.25 to 2.31)	.56	0.06 (–0.23 to 0.36)	0.64 (0.37 to 0.91)	0.88 (0.59 to 1.15)
	3 months	7.67 (6.42)	8.03 (6.78)	0.59 (–1.56 to 2.73)	.59	–0.05 (–0.42 to 0.32)	0.80 (0.47 to 1.12)	0.89 (0.57 to 1.21)
	6 months	7.89 (7.99)	9.88 (7.72)	0.32 (–1.91 to 2.56)	.78	–0.25 (–0.64 to 0.14)	0.74 (0.42 to 1.07)	0.64 (0.31 to 0.98)
**Positive automatic thoughts**								
	Baseline	1.85 (0.55)	1.79 (0.52)	N/A	N/A	N/A	N/A	N/A
	Post	2.4*0* (0.71)	2.33 (0.67)	0.01 (–0.18 to 0.2)	.93	0.1 (–0.19 to 0.39)	–0.88 (–1.16 to –0.6)	–0.93 (–1.21 to –0.65)
	3 months	2.49 (0.67)	2.44 (0.84)	–0.1 (–0.32 to 0.12)	.38	0.07 (–0.3 to 0.44)	–1.08 (–1.41 to –0.75)	–1.03 (–1.36 to –0.71)
	6 months	2.46 (0.85)	2.15 (0.71)	0.08 (–0.16 to 0.31)	.52	0.47 (0.08 to 0.86)	–0.93 (–1.26 to –0.6)	–0.63 (–0.96 to –0.29)
**Negative automatic thoughts**								
	Baseline	2.55 (0.88)	2.65 (0.85)	N/A	N/A	N/A	N/A	N/A
	Post	1.85 (0.78)	1.8*9* (0.6*3*)	–0.02 (–0.22 to 0.18)	.86	–0.04 (–0.33 to 0.25)	0.83 (0.55 to 1.1)	1 (0.72 to 1.28)
	3 months	1.74 (0.68)	1.82 (0.71)	–0.03 (–0.27 to 0.21)	.81	–0.09 (–0.46 to 0.28)	0.97 (0.64 to 1.29)	1.02 (0.7 to 1.34)
	6 months	1.75 (0.78)	2.05 (0.93)	–0.15 (–0.4 to 0.1)	.24	–0.38 (–0.77 to 0.01)	0.93 (0.6 to 1.26)	0.68 (0.34 to 1.02)
**Positive emotions**								
	Baseline	2.72 (0.54)	2.65 (0.54)	N/A	N/A	N/A	N/A	N/A
	Post	3.26 (0.69)	3.2*7* (0.62)	–0.03 (–0.22 to 0.16)	.87	–0.04 (–0.33 to 0.25)	–0.89 (–1.17 to –0.61)	–1.1 (–1.38 to –0.81)
	3 months	3.26 (0.75)	3.33 (0.81)	–0.1 (–0.33 to 0.13)	.93	–0.12 (–0.49 to 0.25)	–0.89 (–1.21 to –0.56)	–1.07 (–1.39 to –0.74)
	6 months	3.24 (0.75)	2.97 (0.72)	0.12 (–0.11 to 0.36)	.38	0.4 (0.01 to 0.79)	–0.85 (–1.18 to –0.52)	–0.54 (–0.88 to –0.21)
**Negative emotions**								
	Baseline	3.73 (0.75)	3.78 (0.73)	N/A	N/A	N/A	N/A	N/A
	Post	2.91 (0.82)	2.9*3* (0.*69*)	–0.02 (–0.23 to 0.2)	.63	–0.02 (–0.31 to 0.28)	1.06 (0.78 to 1.34)	1.21 (0.91 to 1.5)
	3 months	2.78 (0.85)	2.93 (0.73)	–0.07 (–0.32 to 0.18)	.75	–0.19 (–0.56 to 0.18)	1.23 (0.89 to 1.57)	1.17 (0.84 to 1.5)
	6 months	2.86 (0.91)	3.17 (0.88)	–0.1 (–0.36 to 0.16)	.35	–0.39 (–0.78 to 0)	1.09 (0.75 to 1.42)	0.8 (0.46 to 1.13)

^a^iCBT: internet-based cognitive behavioral therapy

^b^BDI-II: Beck Depression Inventory-II.

^c^N/A: not applicable.

^d^PHQ-9: 9-item Patient Health Questionnaire.

^e^GHQ-12: 12-item General Health Questionnaire.

^f^BAI: Beck Anxiety Inventory.

The correction will appear in the online version of the paper on the JMIR Publications website, together with the publication of this correction notice. Because this was made after submission to PubMed, PubMed Central, and other full-text repositories, the corrected article has also been resubmitted to those repositories.
