# Supplementary figures and images for: Culturally Adapted Guided Internet-Based Cognitive Behavioral Therapy for Hong Kong People With Depressive Symptoms: Randomized Controlled Trial
Source: J Med Internet Res. 2025 Feb 25;27:e64303. doi: 10.2196/64303 (PMC11897664; doi:10.2196/64303)

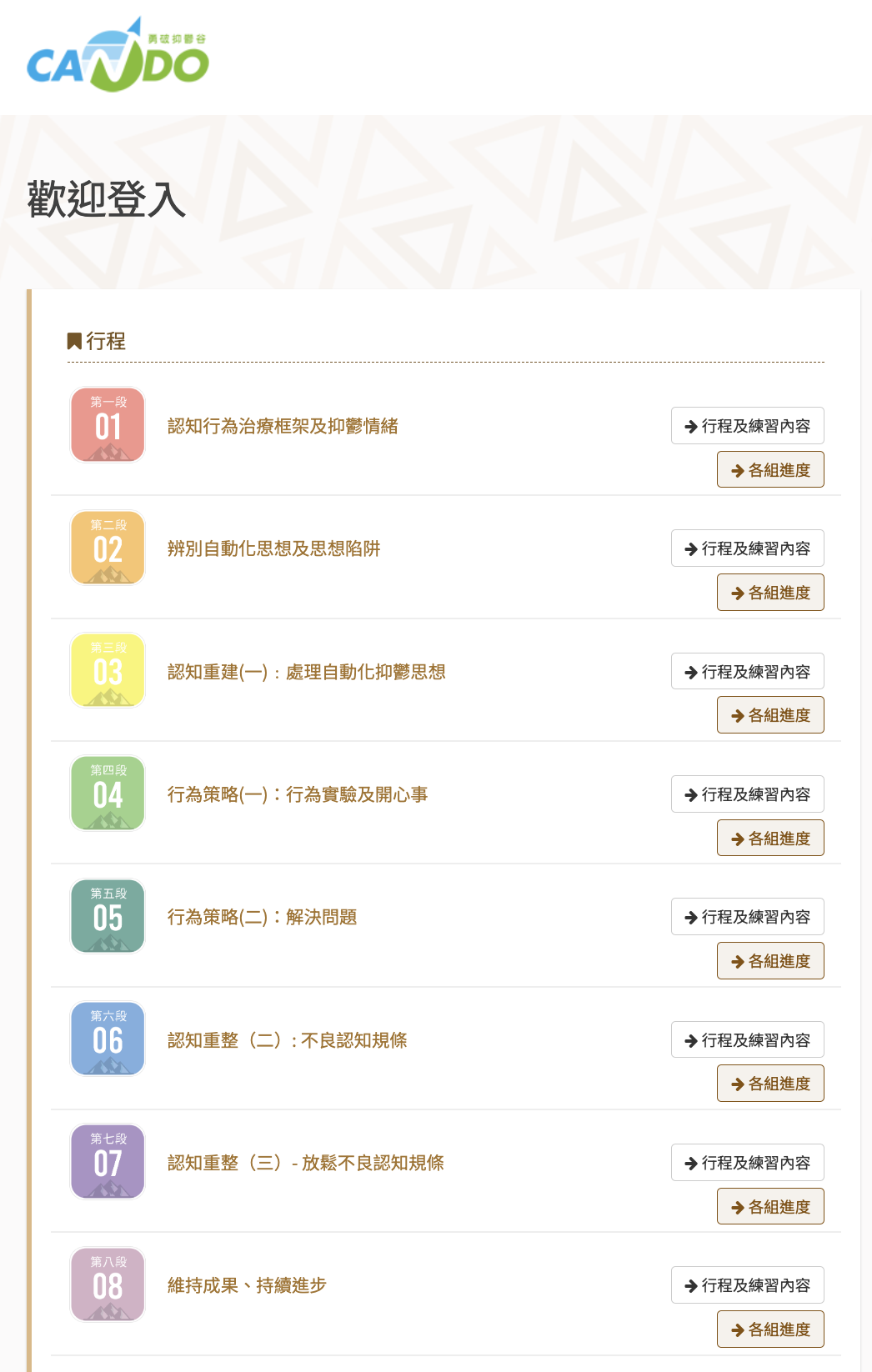


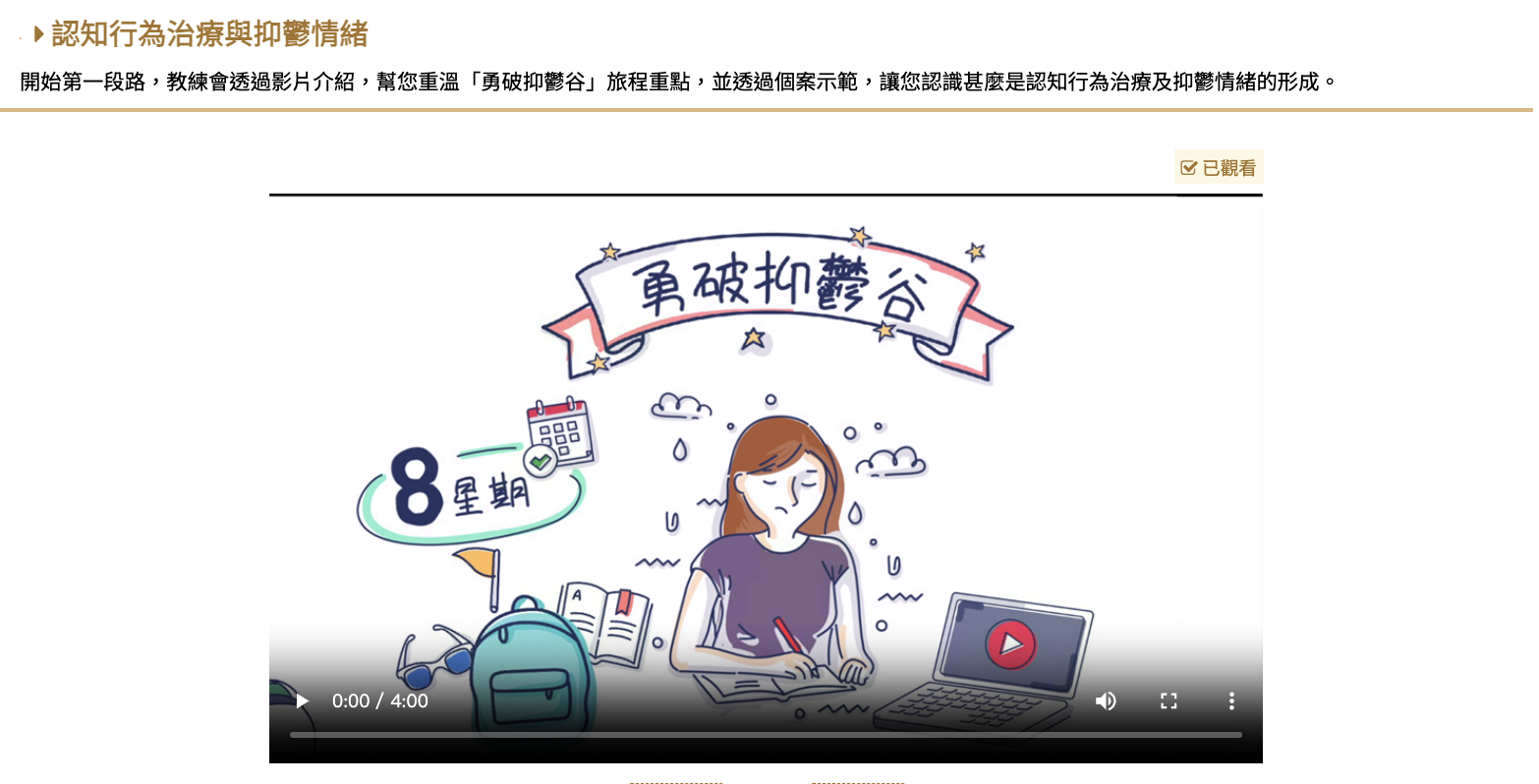


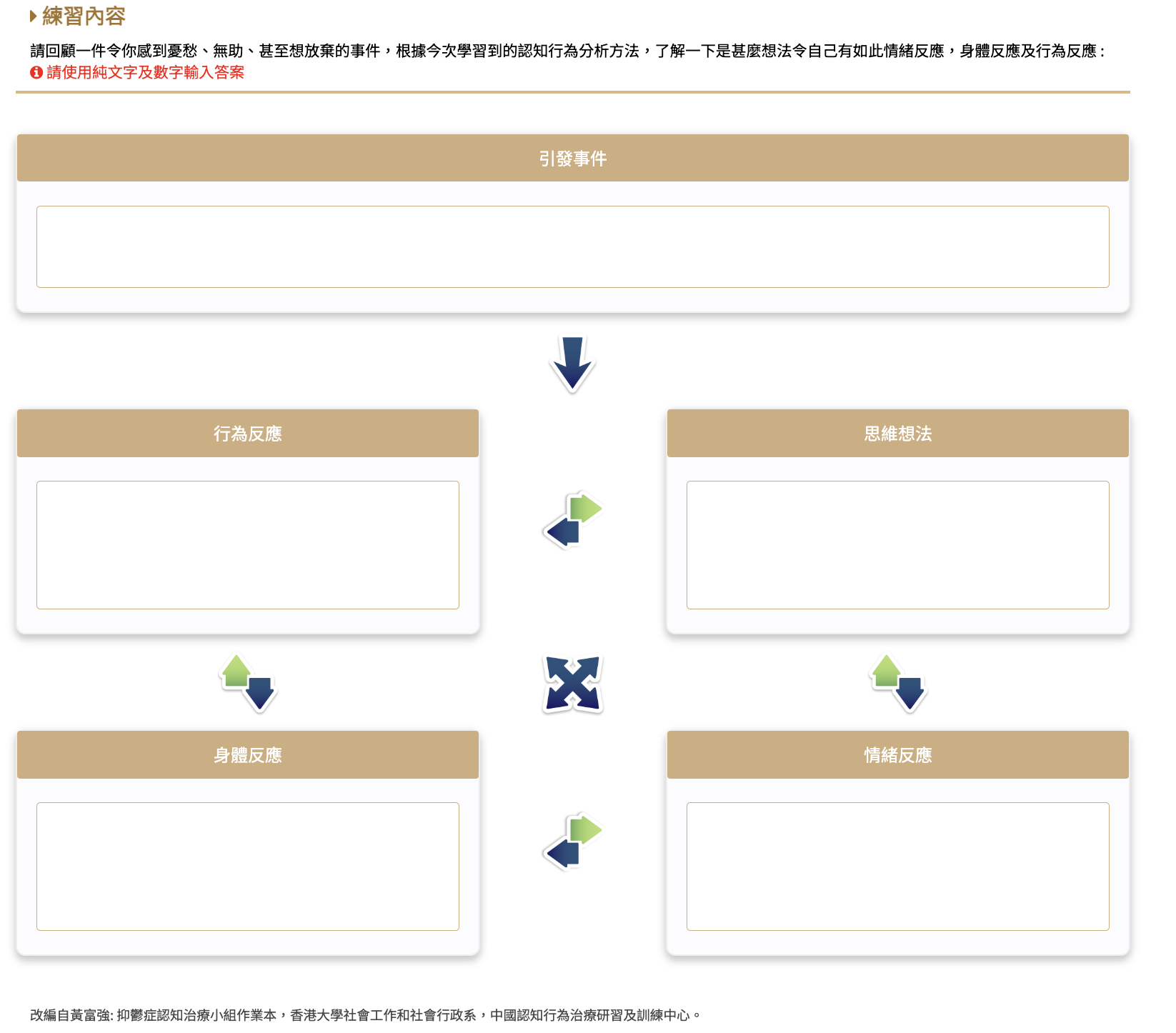

Supplement: Multimedia Appendix 1 [file jmir_v27i1e64303_app1.docx]
