# Supplementary material for: Culturally Adapted Guided Internet-Based Cognitive Behavioral Therapy for Hong Kong People With Depressive Symptoms: Randomized Controlled Trial
Source: J Med Internet Res. 2025 Feb 25;27:e64303. doi: 10.2196/64303 (PMC11897664; doi:10.2196/64303)
Supplement: Multimedia Appendix 2 [file jmir_v27i1e64303_app2.docx]

Supplementary Table S1 *Sensitivity Analysis using ANCOVA and Completer-only Data.*

|  |  | Estimated treatment effects | | | | | | |
| --- | --- | --- | --- | --- | --- | --- | --- | --- |
|  | Web Group | | | |  | App Group | | |
| Outcome | Adj. difference | | p-value | Cohen’s d [95% CI] |  | Adj. difference | p-value | Cohen’s d [95% CI] |
| BDI | -8,18 | | <0.001 | -1.12 [-0.82, -1.41] |  | -8,86 | <0.001 | -1.21 [-0.91, -1.51] |
| PHQ | -3,2 | | <0.001 | -0.82 [-0.49, -1.16] |  | -3,75 | <0.001 | -0.96 [-0.63, -1.29] |
| GHQ | -3,69 | | <0.001 | -1.17 [-0.87, -1.46] |  | -3,94 | <0.001 | -1.24 [-0.94, -1.54] |
| BAI | -2,2 | | 0.01 | -0.41 [-0.13, -0.69] |  | -2,8 | 0.001 | -0.52 [-0.24, -0.81] |
| posAT | 0,39 | | <0.001 | 0.68 [0.97, 0.4] |  | 0,37 | <0.001 | 0.65 [0.94, 0.37] |
| negAT | -0,42 | | <0.001 | -0.67 [-0.38, -0.95] |  | -0,4 | <0.001 | -0.64 [-0.36, -0.93] |
| posEM | 0,34 | | <0.001 | 0.58 [0.86, 0.29] |  | 0,37 | <0.001 | 0.63 [0.91, 0.34] |
| negEM | -0,43 | | <0.001 | -0.67 [-0.38, -0.96] |  | -0,41 | <0.001 | -0.65 [-0.36, -0.93] |

Supplementary Table S2 *Characteristics of participants who completed all eight modules vs non-completers*

| Characteristics | Completers  (N = 154 | Non-completers  (N = 116) | OR (p) | χ2 (p) |
| --- | --- | --- | --- | --- |
| Age, *M (SD)* | 39.47 (11.33) | 35.29 (10.69) | 1.04 (<.01)* |  |
| Female, *n (%)* | 120 (77.9) | 86 (74.1) |  | 0.34 (.56) |
| Education, *n (%)* |  |  |  | 1.2 (.27) |
| Primary school or below | 0 (0.0) | 1 (0.9) |  |  |
| Secondary school | 30 (19.5) | 30 (25.9) |  |  |
| University or above | 121 (78.6) | 84 (72.4) |  |  |
| Marital status, *n* *(%)* |  |  |  | 1.88 (.39) |
| Single | 82 (53.2) | 68 (58.6) |  |  |
| Married | 55 (35.7) | 32 (27.6) |  |  |
| Other | 11 (7.1) | 10 (8.6) |  |  |
| App group, (*%*) | 75 (48.7) | 56 (48.3) |  | 0 (1) |
| Diagnosis, *n* *(%)* |  |  |  | 0.13 (.94) |
| Depression | 34 (22.1) | 21 (18.1) |  |  |
| Anxiety | 13 (8.4) | 7 (6.0) |  |  |
| Other | 18 (11.7) | 12 (10.3) |  |  |

Notes: * p <.01.

Abbreviations: p = p value, χ2 = Pearson Chi-Square, OR = Odds Ratio.

Supplementary Table S3 *Characteristics of post measurement completer vs non-completer participants*

| Characteristics | Completers  (N = 181 | Non-completers  (N = 86) | OR (p) | χ2 (p) |
| --- | --- | --- | --- | --- |
| Age, *M (SD)* | 35.73 (10.65) | 38.64 (11.42) | 1.02 (.047) |  |
| Female, *n (%)* | 64 (71.9) | 142 (78.5) |  | 1.07 (.3) |
| Education, *n (%)* |  |  |  | 3.28 (.07) |
| Primary school or below | 1 ( 1.1) | 0 ( 0.0) |  |  |
| Secondary school | 26 (29.2) | 34 (18.8) |  |  |
| University or above | 61 (68.5) | 144 (79.6) |  |  |
| Marital status, *n* *(%)* |  |  |  | 1.15 (.56) |
| Single | 52 (58.4) | 98 (54.1) |  |  |
| Married | 25 (28.1) | 62 (34.3) |  |  |
| Other | 8 ( 9.0) | 13 ( 7.2) |  |  |
| App group, (*%*) | 40 (44.9) | 91 (50.3) |  | 0.48 (.49) |
| Diagnosis, *n* *(%)* |  |  |  | 1.42 (.49) |
| Depression | 19 (21.3) | 36 (19.9) |  |  |
| Anxiety | 5 ( 5.6) | 15 ( 8.3) |  |  |
| Other | 7 ( 7.9) | 23 (12.7) |  |  |

Abbreviations: p = p value, χ2 = Pearson Chi-Square, OR = Odds Ratio.

| Supplementary Table S4: Estimated effect over time for the waitlist condition when transitioning to active group, using the second pre-measurement as baseline. | | | | |
| --- | --- | --- | --- | --- |
| Measure and time | Unadjusted means (SD) | Within-group adjusted difference (95% CI) | p value | Within-group effect size (95% CI) |
| **BDI** |  |  |  |  |
| Pre 1 | 22.11 (9.08) |  |  |  |
| Post 1/Pre 2 | 18.02 (9.02) |  |  |  |
| Post 2 | 10.32 (9.1) | 6.82 (8.78, 4.86) | < 0.001 | 0.85 (0.53, 1.17) |
| 3 months | 10.33 (7.5) | 7.05 (9.68, 4.42) | < 0.001 | 0.88 (0.46, 1.29) |
| 6 months | 10.82 (8.33) | 6.1 (8.62, 3.57) | < 0.001 | 0.81 (0.41, 1.21) |
| **PHQ** |  |  |  |  |
| Pre 1 | 11.05 (4.44) |  |  |  |
| Post 1/Pre 2 | 8.99 (4.58) |  |  |  |
| Post | 5.71 (4.46) | 2.99 (4.29, 1.7) | < 0.001 | 0.72 (0.4, 1.04) |
| 3 months | 5.9 (3.35) | 2.98 (4.8, 1.17) | 0.012 | 0.71 (0.3, 1.12) |
| 6 months | 6.33 (3.76) | 2.39 (4.03, 0.75) | 0.015 | 0.6 (0.21, 1) |
| **GHQ** |  |  |  |  |
| Pre 1 | 7.63 (3.34) |  |  |  |
| Post 1/Pre 2 | 5.35 (3.85) |  |  |  |
| Post | 1.37 (2.78) | 3.85 (4.82, 2.87) | < 0.001 | 1.14 (0.8, 1.47) |
| 3 months | 2.03 (2.28) | 3.29 (4.59, 2) | < 0.001 | 0.93 (0.51, 1.34) |
| 6 months | 2.27 (2.79) | 3.03 (4.27, 1.79) | < 0.001 | 0.85 (0.44, 1.24) |
| **BAI** |  |  |  |  |
| Pre 1 | 14.2 (9.22) |  |  |  |
| Post 1/Pre 2 | 11.09 (7.49) |  |  |  |
| Post | 7.97 (6.19) | 2.74 (4.2, 1.28) | 0.003 | 0.44 (0.13, 0.75) |
| 3 months | 7.27 (5.44) | 3.1 (5.05, 1.14) | 0.013 | 0.54 (0.13, 0.94) |
| 6 months | 8.18 (6.81) | 2.35 (4.23, 0.47) | 0.016 | 0.4 (0, 0.79) |
| **posAT** |  |  |  |  |
| Pre 1 | 1.82 (0.49) |  |  |  |
| Post 1/Pre 2 | 1.98 (0.58) |  |  |  |
| Post | 2.41 (0.81) | -0.37 (-0.22, -0.52) | < 0.001 | -0.64 (-0.96, -0.33) |
| 3 months | 2.36 (0.74) | -0.28 (-0.07, -0.48) | 0.016 | -0.61 (-1.02, -0.2) |
| 6 months | 2.45 (0.81) | -0.38 (-0.18, -0.57) | 0.003 | -0.72 (-1.12, -0.33) |
| **negAT** |  |  |  |  |
| Pre 1 | 2.51 (0.79) |  |  |  |
| Post 1/Pre 2 | 2.29 (0.8) |  |  |  |
| Post | 1.88 (0.74) | 0.36 (0.55, 0.18) | 0.002 | 0.52 (0.21, 0.84) |
| 3 months | 1.86 (0.64) | 0.41 (0.66, 0.16) | 0.011 | 0.56 (0.15, 0.97) |
| 6 months | 1.85 (0.9) | 0.36 (0.59, 0.12) | 0.015 | 0.53 (0.14, 0.93) |
| **posEM** |  |  |  |  |
| Pre 1 | 2.71 (0.54) |  |  |  |
| Post 1/Pre 2 | 2.89 (0.68) |  |  |  |
| Post | 3.3 (0.76) | -0.37 (-0.22, -0.53) | < 0.001 | -0.58 (-0.89, -0.27) |
| 3 months | 3.31 (0.77) | -0.34 (-0.13, -0.55) | 0.012 | -0.6 (-1, -0.19) |
| 6 months | 3.23 (0.73) | -0.31 (-0.11, -0.51) | 0.013 | -0.5 (-0.89, -0.1) |
| **negEM** |  |  |  |  |
| Pre 1 | 3.69 (0.72) |  |  |  |
| Post 1/Pre 2 | 3.33 (0.74) |  |  |  |
| Post | 2.94 (0.74) | 0.37 (0.54, 0.2) | < 0.001 | 0.53 (0.21, 0.84) |
| 3 months | 2.83 (0.72) | 0.51 (0.74, 0.29) | < 0.001 | 0.68 (0.27, 1.09) |
| 6 months | 2.88 (0.79) | 0.42 (0.63, 0.2) | 0.003 | 0.6 (0.2, 0.99) |
| Abbreviations: BDI = Beck Depression Inventory, PHQ-9 = Patient Health Questionnaire, BAI = Beck Anxiety Inventory, GHQ = General Health Questionnaire, posAT = Positive Automatic Thoughts, negAT = Negative Automatic Thoughts, posEM = Positive Emotion, negAT = Negative Emotion, SD = Standard Deviation, CI = Confidence Interval, d = Cohens’ d. | | | | |
